# Transcutaneous Tibial Nerve Stimulation for Pain Management in Women with Primary Dysmenorrhea: A Randomized Clinical Trial

**DOI:** 10.3390/biomedicines12092093

**Published:** 2024-09-13

**Authors:** Marta Correyero-León, Javier Calvo-Rodrigo, Jorge Juan Alvarado-Omenat, Rocío Llamas-Ramos, Mª Consuelo Martínez-Terol, Inés Llamas-Ramos

**Affiliations:** 1 CRA La Villa, Calle Calvario 13, 47300 Peñafiel, Spain; marta.correyero.leon@gmail.com; 2 CEE Fuenteminaya, Calle Padre Janáriz, 11, 09400 Aranda de Duero, Spain; calvorodrigoj@gmail.com; 3 FisioSport Salamanca, 12 de Octubre, n_ 2, 37008 Salamanca, Spain; jjao@usal.es; 4 Department of Nursing and Physiotherapy, Universidad de Salamanca, Avda, Donantes de Sangre s/n, 37007 Salamanca, Spain; inesllamas@usal.es; 5 Institute of Biomedical Research of Salamanca (IBSAL), 37007 Salamanca, Spain; 6 Centro de Rehabilitación Tenerías, Plaza Tenerías 5, 47006 Valladolid, Spain; 7 University Hospital of Salamanca, Health Service of Castile and Leon (SACyL), P.° de San Vicente, 182, 37007 Salamanca, Spain

**Keywords:** primary dysmenorrhea, posterior tibial nerve stimulation, pain, physiotherapy, randomized clinical trial

## Abstract

Primary dysmenorrhea is considered one of the main causes of pelvic pain during a woman’s childbearing years, resulting in poor quality of life. The objective was to evaluate the effectiveness of transcutaneous tibial nerve stimulation (TTNS) in painful symptomatology improvement and non-steroidal anti-inflammatory drug (NSAID) intake reduction in women with primary dysmenorrhea (PD) compared with a control group in the short, medium, and long terms. A single-blind, controlled clinical trial was developed. Participants were randomized to the experimental (TTNS) and control group (sham TTNS). Both groups received 12-weekly 30-min sessions with a NeuroTrac^TM^ PelviTone electrostimulation device. The intensity and severity of pain and non-steroidal anti-inflammatory drug (NSAID) intake were evaluated in the short-term (after treatment), medium-term (1–3 months), and long-term (6 months). A total of 61 participants were randomized, with a split of 31 (experimental group) and 30 (control group), but 55 participants completed the study and were analyzed. Statistically significant differences between both groups in the maximum pain intensity decrease (F = 4.88, *p* = 0.0043) measured with the visual analogue scale, as well as NSAID intake decrease (F = 4.68, *p* = 0.011) and days of their ingestion (F = 4.57, *p* = 0.012) occurred in the short term. Furthermore, significant decreases in the total number of NSAIDs ingested during the cycle (F = 3.82, *p* = 0.011) and the number of days on which patients ingested NSAIDs (F = 3.59, *p* = 0.015) in the medium–long term occurred. TTNS could be an effective and safe strategy to reduce pain caused by PD, which could reduce or complement the use of pharmacological techniques and other more invasive methods.

## 1. Introduction

Primary dysmenorrhea (PD), defined as pain present during menstruation in the absence of pelvic disease, is one of the main causes of pelvic pain during a woman’s childbearing years [1,2,3], with a prevalence of between 45% and 95% of the female population at this stage [2,3,4]. This cramping pain is located in the lower abdominal and suprapubic area, and may radiate to the back and thighs, usually lasting from 48 to 72 h, being more intense during the first and second days of menstruation [2,3,4]. Regarding its etiology, the most accepted theory suggests that the pain is caused by elevated levels of prostaglandins (PGs) released by the endometrium into the menstrual fluid (especially PGF2α and PGE2). Physiologically, these PGs induce vasoconstriction and myometrial contractions, promoting the expulsion of menstrual flow from the uterine cavity. However, when these PGs are present in high quantities, they cause uterine hypercontractility, leading to hypoxia and ischemia of the endometrial mucosa. Both the myometrium of dysmenorrheic women and those without pain are sensitive to PGF2α; the difference lies in that the amount of PGF2α and the difference between progesterone and prostaglandin levels are much higher in women with pain [1,2,3,5]. Along with elevated prostaglandin levels, dysmenorrheic women have higher levels of uterine activity during menstruation compared with asymptomatic women. This results in intrauterine pressures that can exceed 400 mm of mercury (mmHg) and basal intrauterine pressures that exceed 80 mmHg (normal basal pressure is around 10–20 mmHg). Additionally, the frequency of uterine contractions and uncoordinated uterine contractions are more numerous in women with dysmenorrhea. These abnormally strong contractions are associated with reduced uterine blood flow, resulting in myometrial ischemia and, consequently, pain [3,6]. Some studies suggest that these women tend to have a greater central sensitization to pain [1,3,7,8]. The main treatment for PD consists of non-steroidal anti-inflammatory drugs (NSAIDs) or hormonal therapy, which have proven to be effective in many women, although there is a percentage of women who do not respond to the treatments or do not tolerate their intake [2,3]. For this reason, different conservative non-pharmacological treatments, such as transcutaneous electrical nerve stimulation (TENS) [2,9,10] kinesiotaping [10,11] or physical exercise, have been proposed in the literature [10,12,13]; approaches that are recommended as complementary therapies to reduce menstrual pain in the short term with a low quality of evidence [2,10].

The present study proposes the use of transcutaneous tibial nerve stimulation (TTNS), which consists of the stimulation of the posterior tibial nerve in the superomedial region of the ankle. The hypogastric sympathetic plexus (L4–L5) and the pelvic parasympathetic plexus (S2–S4) have the same medullar level as the posterior tibial nerve (L4–S3) [14]. Thus, inhibitory and excitatory impulses that control the function of the pelvic viscera at the level of the spinal cord can be rebalanced with their stimulation [15]. 

This technique began to be applied by McGuire et al. [16] in 1983 and by Stoller in 1999 [17] for the treatment of overactive bladder. Since then, the technique has evolved in its two application modalities (transcutaneous and percutaneous), and positive results have been found in the reduction of symptoms of pathologies such as overactive bladder [18,19,20], fecal incontinence [21,22], and chronic pelvic pain [23]. In the literature, it is proposed that the decrease in pain is due to factors such as the inhibition of the afferent A delta and C fibers by the stimulation of the somatic fibers (gate control), an increase in endorphins at the spinal level, or a decrease in c-fos expression in the central nervous system [24]. In addition to these theories, we hypothesized that the stimulation of the tibialis posterior will rebalance the contraction signals of the uterine myometrium, reducing uterine hypercontractility and thus reducing menstrual pain. 

However, there are no specific studies in the literature that have treated PD by TTNS, so the aim of the present study was to verify whether TTNS improves painful symptomatology and reduces NSAID intake versus a control group of women with PD in the short, medium, and long term, without causing adverse effects.

## 2. Materials and Methods

### 2.1. Study Design

A randomized, single-blind, controlled clinical trial was conducted to evaluate the efficacy and safety of TTNS in patients with PD. This study took place in a private physiotherapy center (Centro de Rehabilitación Tenerías) in the province of Valladolid, Spain. 

On 12 December 2019, the Ethics Committee for Drug Research, Valladolid East Health Area, of the Hospital Clínico Universitario de Valladolid, located at Hospital Clínico Universitario de Valladolid, Av. Ramón y Cajal, 3, 47003 Valladolid (Spain) approved this study (acceptance code CASVE-NM-19-423). Helsinki and CONSORT guidelines were followed and all participants were informed and signed an informed consent to participate The registration number in ClinicalTrials.gov is ID: NCT04896814; https://clinicaltrials.gov/study/NCT04896814 (accessed on 21 August 2024).

### 2.2. Sample

To participate in this study, participants were required to follow some inclusion criteria. They were: being women between 18 and 43 years of age, to have regular menstrual cycles, and to suffer pain located in the suprapubic area, abdomen, lower lumbar area, perineum, and/or medial aspect of the thighs during at least half of their annual menstrual cycles and/or in the last 3 cycles and more than 4 points of the visual analogue scale (VAS). Women were excluded if they were taking oral contraceptives or had an intrauterine device implanted, were diagnosed with gynecological pathology, had undergone surgery or childbirth in the last 6 months, pregnant women, with uncorrected coagulopathies, severe comorbid disorder, cancer in the last 5 years or at present, presence of erosions on the inner aspect of the ankle, severe mental disorders, neuropathies affecting the lower limb, and those women who had received physiotherapy treatment for PD one month before the start of this study. 

The sample size was calculated using a G-Power program and based on the data collected in previous investigations [25,26,27,28,29]. An alpha risk of 0.05 was accepted and a beta risk of 0.2 was considered in a bilateral contrast. For this purpose, 27 subjects in the first group and 27 in the second group were necessary to detect a difference equal to or greater than 1.3 in peak VAS. A common standard deviation of 1.6 was assumed and a loss-to-follow-up rate of 10% was estimated.

### 2.3. Randomization

All participants included in this study were randomly assigned to the experimental or control group, with a 1:1 ratio. Randomization was carried out using the Random Number Generator Pro software (version 1.76). The distribution of the groups was hidden from the researcher, since she did not know to which group each subject would be assigned when the decision was made to include them in the study. The participants were unaware of their group assignment.

### 2.4. Procedure

This study consisted of 4 phases: a first interview, an initial evaluation phase with a duration of 2 months, an intervention phase, and a reevaluation phase. 

Recruitment and randomization of patients were performed from 25 May 2021, to 31 May 2021. Baseline assessment was performed in the months of June and July 2021 and interventions were performed from 1 August to 31 October 2021. Short- and medium–long-term monitoring was conducted from 1 November 2021, to 30 April 2022.

In the first phase, the selected participants attended an initial interview with the researcher, lasting for approximately 30 min, during which they were informed about the characteristics of this study. They signed the informed consent form if they wished to participate in the study and filled out a medical history form. In the second phase of the initial assessment, lasting for 2 months, several questionnaires were completed over two consecutive menstrual periods. These questionnaires were supplied to the participants during the first interview and were self-administered during the following two menstrual periods. In the third intervention phase, lasting for 3 months, a weekly intervention session was conducted. During this phase, the participants continued to fill out the self-administered questionnaires on a monthly basis.

**Figure 1:**
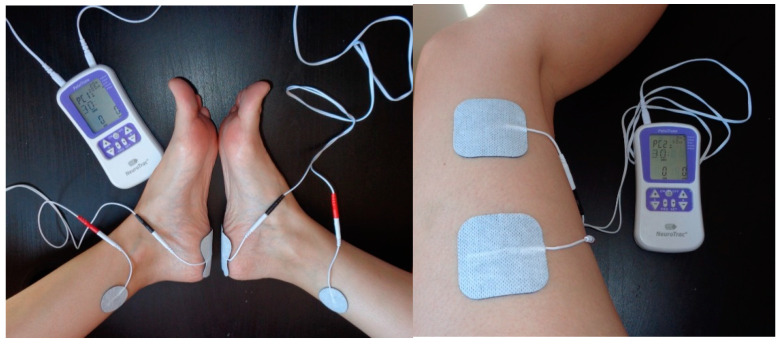
Electrode location for TTNS Intervention (left: experimental group and right: control group).

The procedure of this study will be established in two arms. The experimental group received a TTNS intervention, and the control group received a sham current outside the posterior tibial nerve territory. Both groups received a weekly 30-min session for 12 weeks. The NeuroTracTM PelviTone electrostimulation device was used to treat the patients (Figure 1). 

The experimental group received a symmetrical biphasic current in continuous mode (20 Hz and 200 µs) with the patient in supine position for 30 min. The knees were flexed and abducted at 90°. Stimulation was applied to both legs. Two electrodes (32 mm of diameter) were placed cranial to the internal malleolus of each leg and the other electrodes (50 × 50 mm) were placed at the ipsilateral calcaneus. The stimulation was regulated between 1 and 30 Ma [30].

The control group received a discontinuous current at 2 Hz frequency and a pulsed frequency of 50 s (2 s of work and 10 s of pause) as a simulated current in the same position and for the same time. Two 50 × 50 electrodes were placed in the external face of the thigh (only one leg) [30]. 

In the fourth re-evaluation phase, lasting for 6 months, the participants completed the same questionnaires 1 month, 3 months, and 6 months after the intervention ended.

The intervention phases of the study, the description of the procedures, and the variables evaluated are detailed in the publication by Correyero-León et al. [30].

### 2.5. Data Collection and Outcomes

Self-administered questionnaires were used to collect the data and SPSS v26 software (IBM Corporation, Armonk, NY, USA) was used for the randomization process.

During the study implementation, several variables were collected. VAS was selected to collect the maximum and mean pain intensity and pain duration [31,32]. The patient used the maximum pain that she experienced each day of her menstruation to fill in the scale. To evaluate the pain severity, McGill’s Short Questionnaire (SF-MPQ^®^) was selected. The higher punctuation of the SF-MPQ^®^ score, the greater the pain severity [33,34]. The day of maximum pain of her menstruation was when the patient filled in this questionnaire. Another variable collected was the number of NSAIDs taken by the patient; they were recorded in a diary. The information registered in this diary by the patients were each NSAID’s type and amount taken (all days of the menstruation) and even the pain relief obtained with each dose.

These data were collected in an initial phase with a duration of two months to observe the baseline status of the patients, once a month during the intervention (short term) and 1 month, 3 months (medium term), and 6 months (long term) after the end of the intervention.

Adverse reactions due to the treatment were collected, and a short questionnaire created by the investigator was filled in by the participants after each treatment session and 11 months after the onset (follow up).

### 2.6. Statistical Analyses

Statistical analysis was performed using SPSS v26 software (IBM Corporation, Armonk, NY, USA). Quantitative variables were represented as the mean and standard deviation and qualitative variables were calculated using the number and percentages. The Shapiro–Wilk test was used to test the normality. Student’s T-test was selected to compare both groups. The effect of the intervention between groups was also analyzed with a repeated-measures two-factor analysis of variance (rANOVA) to examine disparities in outcomes over time, considering baseline and post-intervention as one factor within each subject, and the group (experimental and control) as another factor. A value of *p* < 0.05 will be established as statistically significant.

## 3. Results

**Figure 2:**
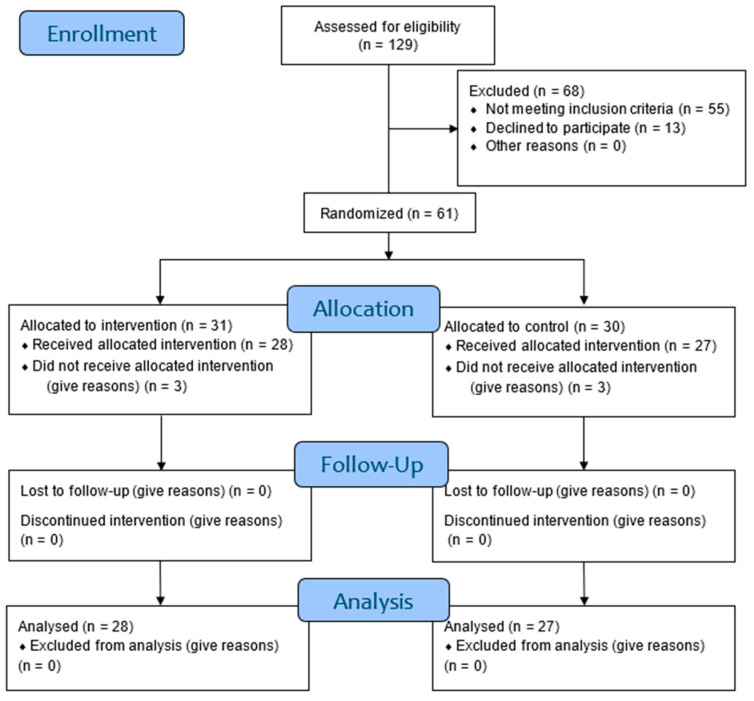
Flow diagram.

Of the 129 participants recruited, a total of 61 participants were randomized, 31 were assigned to the experimental group and 30 to the control group. Of these, four dropped out of the study in the evaluation phase without completing it and two completed phase 2, but left the treatment without completing it (intervention phase). There were no losses in the reassessment phase. A total of 55 participants completed the entire clinical trial and were part of the statistical analysis. No losses were obtained during the phase 4 follow-up. The flow diagram of participant recruitment and follow-up is shown in Figure 2. 

A summary of the baseline characteristics of the study participants is presented in Table 1. The participants were between 18 and 44 years old. The average age was 28.4 (7.1) and 25.3 (5.6) years for the control and experimental groups, respectively. Both groups were similar in baseline demographic characteristics, as well as in their baseline status and age (*p* = 0.078). 

**Figure 3:**
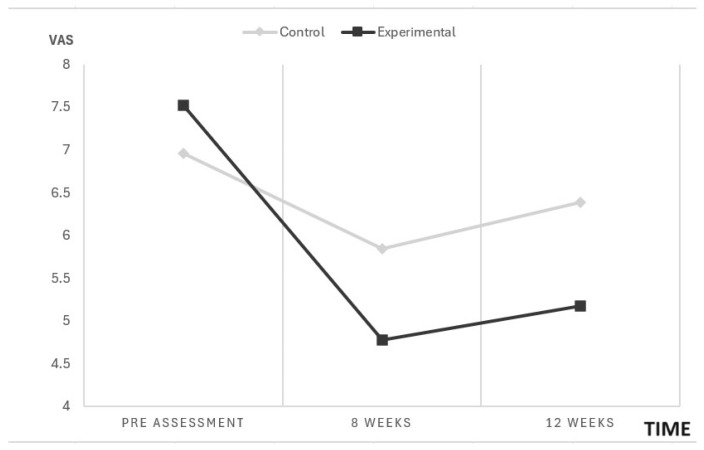
rANOVA for VAS.

After the end of the 12-week intervention, rANOVA (normal sample distribution) showed statistically significant group-by-time interactions for maximum pain intensity measured with the VAS (F = 4.88; *p* = 0.009). Figure 3 shows that, for the experimental group, there were statistically significant differences *p* < 0.001 between the pre-assessment and at 8 and 12 weeks, respectively, but there were no differences between 8 and 12 weeks.

**Figure 4:**
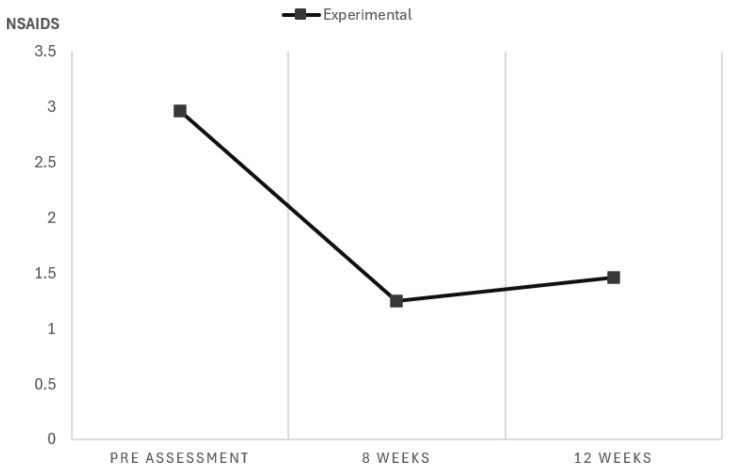
NSAID intake in experimental group.

The total number of NSAIDs ingested during the cycle decreased significantly (F = 4.68, *p* = 0.011), as well as the number of days on which patients ingested NSAIDs (F = 4.57, *p* = 0.012). This variable could not be compared with the control group, although there were statistically significant differences at the beginning. However, the experimental group showed a reduction in the total number of NSAIDs ingested in the experimental group (*p* < 0.001) between the pre-assessment and after 8 and 12 weeks (only in the experimental group) (Figure 4).

Finally, there were no statistically significant differences in the total score of the SF-MPQ^®^ questionnaire (Table 2 and Table 3).

In the medium–long term (re-evaluations at 1, 3, and 6 months after the end of the treatment), the rANOVA showed statistically significant results in the decrease in the total number of NSAIDs ingested during the cycle (F = 3.82, *p* = 0.011). There were statistically significant differences between pre-assessment and 1 month after the end of the treatment in the experimental group; however, there were none for the control group (*p* < 0.009). Additionally, there was a reduction in the number of days in which patients ingested NSAIDs (F = 3.59, *p* = 0.015), with statistically significant differences between pre-assessment and 1, 3, and 6 months after the end of the treatment in the experimental group; however, there were none for the control group (*p* < 0.05).

There was no interaction for the maximum pain intensity (F = 0.101, *p* = 0.959) and we could analyze each of the factors independently. Finally, there were no statistically significant differences in the total score of the SF-MPQ^®^ questionnaire.

No adverse effects related to the technique in the short or medium to long term were described. 

## 4. Discussion

The aim of this study was to investigate the effects of TTNS on pain intensity and NSAID intake. This study compared the effects of TTNS with those who received a placebo intervention.

To the best of our knowledge, this is the first randomized controlled clinical trial study to investigate the effects of TTNS for PD pain relief compared with a control group, with statistically significant results in decreasing short-term maximum and average pain and decreasing NSAID intake in the short, medium, and long terms.

Different publications in the literature have studied other electrotherapy techniques for the treatment of PD, showing greater evidence for the use of TENS [35,36,37], with statistically significant differences in the decrease in VAS values in the short term (after 3 months of treatment), as in the present study. In addition, one study observed statistically significant improvements in the decrease in NSAID intake in the short term [35] whereas, in the present work, these improvements were estimated up to 6 months after the intervention. The studies reviewed did not evaluate medium- and long-term results. It is therefore possible to understand the trend toward a decrease in pain in the medium and long term, although no statistically significant differences were found between groups. 

On the other hand, it was found that posterior tibial nerve stimulation, in its two modalities (transcutaneous and percutaneous), was used to treat other pathologies that present with chronic pelvic pain (CPP), observing a decrease in pain in the short term [24,38,39,40], although only one article evaluated long-term improvements, with the decrease in pain being maintained at 6 months after treatment [40], which highlights the importance of the long-term evaluation of the present work. The treatment protocols consisted of the same number of sessions, parameters, and time as in the present study, although these studies included women and men with PCC and most of them performed the technique unilaterally percutaneously, whereas in the present study, it was decided to perform the technique transcutaneously and bilaterally, with the aim of optimizing the results and reducing patient discomfort, given that it has been observed that both strategies obtain the same results [41,42].

It has been hypothesized that the positive results obtained in this study regarding pain relief and reduction in NSAID intake may be related to the fact that neuromodulation through the posterior tibial nerve achieved modulation of uterine contractions, which consequently reduced the pain. Although statistically significant results were not obtained in the reduction of pain intensity in the medium to long term, a trend in this direction is observed. However, a consistent reduction in NSAID intake over time was noted, which may indicate that participants experienced less pain and therefore required less medication, suggesting that the effects of the technique were sustained over time. In this regard, it would be interesting to continue applying the technique beyond the 3-month treatment period, with reinforcement sessions to maintain these results. Therefore, it could be considered that the use of this technique does not have only a temporary effect, but may have an impact in the short and medium term for the participants. 

This study has several limitations, such as the lack of blinding of the investigator administering the treatment, the simultaneous use of NSAIDs with the intervention, and the focus of the treatment on alleviating and reducing pain without addressing the underlying cause, as there are no tools available to measure the rate of prostaglandins or uterine contractility.

It seems that these improvements achieved in the reduction of pain would be related to the stimulation produced on the posterior tibial nerve, and, therefore, on the pelvic nerves, which would modulate nerve transmission and, therefore, uterine hypercontractility, in addition to providing pain relief according to the gate theory, among others.

## 5. Conclusions

The results of this study suggest that TTNS could be an effective and safe strategy in conjunction with NSAIDs to reduce pain caused by PD, which could reduce or complement the use of pharmacological techniques and other more invasive methods. Future studies with a larger sample size are needed to confirm these benefits in the medium to long term, as well as comparative studies to delimit the greater efficacy between some techniques and others.

## Figures and Tables

**Table 1:** Baseline characteristics of the study population.

	Control Group	Experimental Group
Age (years)	28.4 (7.1)	25.3 (5.6)
BMI (kg/cm^2^)	22.14 (3.22)	22.39 (2.85)
NSAIDs Intake n (%)		
No	1 (3.7)	1 (3.6)
Yes	26 (96.3)	27 (96.4)
NSAIDs Relief n (%)		
No	1 (3.7)	0 (0.0)
Yes	19 (70.4)	20 (71.4)
Occasionally	7 (25.9)	8 (28.6)
Children n (%)		
0	23 (85.2)	26 (92.9)
1	2 (7.4)	1 (3.6)
2	1 (3.7)	1 (3.6)
3	1 (3.7)	0 (0.0)
Tobacco n (%)		
No	23 (85.2)	22 (78.6)
Yes	2 (7.4)	2 (7.1)
Occasionally	1 (3.7)	2 (7.1)
Former smoker	1 (3.7)	2 (7.1)
Alcohol n (%)		
No	2 (7.4)	3 (10.7)
Yes	2 (7.4)	1 (3.6)
Occasionally	23 (85.2)	24 (85.7)
Physical Activity n (%)		
No	3 (11.1%)	5 (17.9)
Si	16 (59.6)	17 (60.7)
Occasionally	8 (29.6)	6 (21.4)
Cycle Duration n (%)		
21–27 days	6 (22.2)	4 (14.3)
28–31 days	16 (59.3)	21 (75.0)
32–35 days	5 (18.5)	3 (10.7)
Menstruation Duration n (%)		
1–3 days	1 (3.7)	6 (21.4)
4–6 days	23 (85.2)	19 (67.9)
7–9 days	3 (11.1)	3 (10.7)
Abdominal Pain n (%)		
No	12 (44.4)	9 (32.1)
Yes	15 (55.6)	19 (67.9)
Suprapubic Pain n (%)		
No	2 (7.4)	9 (32.1)
Yes	25 (92.6)	19 (67.9)
Low back Pain n (%)		
No	6 (22.2)	9 (32.1)
Yes	21 (77.8)	19 (67.9)
Medial thigh Pain n (%)		
No	23 (85.2)	25 (89.3)
Yes	4 (14.8)	3 (10.7)
Perineal Pain n (%)		
No	21 (77.8)	22 (78.6)
Yes	6 (22.2)	6 (21.4)

**Table 2:** Mean values obtained in the pre-treatment and post-treatment evaluations of the aspects examined by the VAS, SF-MPQ^®^, and NSAID intake, in the short term.

	Pre-Assessment		8 Weeks		12 Weeks (3 Months)		rANOVA		Mean Difference between Groups at 12 Weeks (95% CI)
Variable	Mean (± SD)		Mean (± SD)		Mean (± SD)		F	*p* value	
	EG (n = 28)	CG (n = 27)	EG (n = 28)	CG (n = 27)	EG (n = 28)	CG (n = 27)	EG (n = 28) vs. CG (n = 27)		
Maximum pain intensity (VAS)	7.52 (1.29)	6.96 (1.72)	4.78 (2.52)	5.85 (2.49)	5.18 (2.47)	6.39 (2.24)	4.88	0.003 *	1.21 (−0.07–2.49)
Pain intensity (SF-MPQ^®^ total)	18.93 (7.51)	18.93 (10.53)	12.14 (9.01)	14.96 (10.96)	13.11 (10.16)	17.3 (12.17)	1.15	0.32	4.19 (−1.87–10.20)
Number of NSAIDs used during cycle	2.96 (2.52)	1.52 (1.78)	1.25 (1.62)	1.30 (1.73)	1.46 (2.15)	1.33 (2.22)	4.68	0.011 *	−0.131 (−1.31–1.05)
Number of days of NSAID intake	1.93 (1.38)	1.11 (1.19)	0.93 (1.08)	1.04 (1.37)	0.96 (1.37)	1 (1.39)	4.57	0.012 *	0.036 (−0.71–0.78)

SD: Standard deviation, EG: experimental group, CG: control group; Intragroup analysis 1: analysis between the data before the intervention and after eight weeks of intervention; Intragroup analysis 2: analysis between the data before the intervention and after twelve weeks of intervention; *: Statistically significant differences; rANOVA: repeated measures two-factor analysis of variance; VAS: visual analogue scale; NSAIDs: non-steroidal anti-inflammatory drugs; SF-MPQ: McGill’s Short Questionnaire.

**Table 3:** Mean values obtained in the pre-treatment and post-treatment evaluations of the aspects examined by the VAS, SF-MPQ^®^ and NSAIDs intake, in the medium-long term.

	Before		4 Months		6 Months		9 Months		rANOVA	
Variable	Mean (± SD)		Mean (± SD)		Mean (± SD)		Mean (± SD)		F	*p* value
	EG (n = 28)	CG (n = 27)	EG (n = 28)	CG (n = 27)	EG (n = 28)	CG (n = 27)	EG (n = 28)	CG (n = 27)	EG (n = 28) vs. CG (n = 27)	
Maximum pain intensity (VAS)	7.52 (1.29)	6.96 (1.72)	5.52 (2.7)	5.29 (2.81)	5.89 (2.8)	5.56 (2.52)	6.04 (2.49)	5.74 (2.74)	0.101	0.959
Pain intensity (SF-MPQ^®^ total)	18.93 (7.51)	18.93 (10.53)	13 (8.95)	15 (12.89)	14.36 (9.93)	16.41 (11.78)	13.71 (9.87)	16.15 (11.84)	0.420	0.0739
Number of NSAIDs used during the cycle	2.96 (2.52)	2.96 (2.52)	1.39 (1.75)	1.30 (1.94)	1.64 (2.09)	1.59 (2.06)	1.68 (2.42)	1.78 (2.17)	3.82	0.011 *
Number of days of NSAID intake	1.93 (1.39)	1.11 (1.19)	1.04 (1.14)	0.93 (1.24)	1.14 (1.18)	1.19 (1.47)	1.04 (1.17)	1.19 (1.24)	3.59	0.015 *

SD: Standard deviation, EG: experimental group, CG: control group; Intragroup analysis 1: analysis between the data before the intervention and the reevaluation at the month after completing the intervention; Intragroup analysis 2: analysis between the data before the intervention and the reevaluation at the three months after completing the intervention; Intragroup analysis 3: analysis between the data before the intervention and the reevaluation at the six months after completing the intervention; *: statistically significant differences; rANOVA: repeated measures two-factor analysis of variance; VAS: visual analogue scale; NSAIDs: non-steroidal anti-inflammatory drugs; SF-MPQ: McGill’s Short Questionnaire.

## Data Availability

The data presented in this study are available on request from the corresponding authors.
